# Evaluation of seasonal antioxidant activity and total phenolic compounds in stems and leaves of some almond (*Prunus amygdalus* L.) varieties

**DOI:** 10.1186/0717-6287-47-9

**Published:** 2014-04-01

**Authors:** Aysel Sivaci, Sevcan Duman

**Affiliations:** Department of Biology, Art and Science Faculty, Adiyaman University, Adiyaman, Turkey; Graduate School of Sciences, Adiyaman University, Adiyaman, Turkey

**Keywords:** Almond, Antioxidant activity, Phenolics, *Prunus amygdalus*, Seasonal changes

## Abstract

**Background:**

This study aimed to determine the seasonal changes of total antioxidant activity and phenolic compounds in samples taken from leaves (April, July, October) and stems (April, July, October, January) of some almond (*Prunus amygdalus* L.) varieties (Nonpareil, Ferragnes and Texas).

**Results:**

It was indicated that antioxidant activity and phenolic compounds in leaves and stems of Nonpareil, Ferragnes and Texas showed seasonal differences. Antioxidant activity IC_50_ of these varieties reached the highest value in April for leaves whereas in October for stems. The highest level of total phenolic compounds was in January for stems while in October for leaves.

**Conclusions:**

These results showed that total antioxidant activity and phenolics in leaves and stems of almond varieties changed according to season and plant organ.

## Background

Climate is a factor which affects agricultural production. Increase of temperature or variations in precipitation ratio affect physiological events in plants [1–3].

Almond belongs to *Rosaceae* family and is an important product due to high commercial value of its fruits. Its fruits are nutritious due to their protein, fat, mineral substance, fibre and vitamin E content [4–10].

Natural products derived from plants are used for health supplements [11]. Antioxidants are compounds which prevent or delay the oxidation of lipids or other molecules by inhibiting the initiation or propagation of oxidative chain reactions have positive effects on human health [12, 13]. Phenolic substances are one of the most widely known substances with their antioxidant characteristics [14, 15]. Phenolic substances are metabolites with different structure and functions, having an aromatic ring containing generally one or more hydroxyl group [16, 17]. Antioxidant effects of phenolic compounds are explained by bonding free radicals, forming chelate with metals and inactivating some enzymes [18]. Various studies carried out on almond cultivars showed that almond fruit and sections have phenolic compounds and antioxidant activity [19–23].

Analysis of previous research on almonds focused on investigating the antioxidant activity and phenolic compounds mostly in fruits, and the changes in stem and leaves have not been studied on seasonal basis. This study will be significant for determining beneficial compounds in different organs of almond varieties, on seasonal basis, the possibility of making use of these organs and explaining the variations in this plant under different climatic conditions. Therefore, this study investigated seasonal total antioxidant activity and total phenolic compounds in leaves and stems of some almond varieties (Nonpareil, Ferragnes and Texas) which are distributed in Adiyaman province of Turkey.

## Results

### Total antioxidant activity

**Figure 1:**
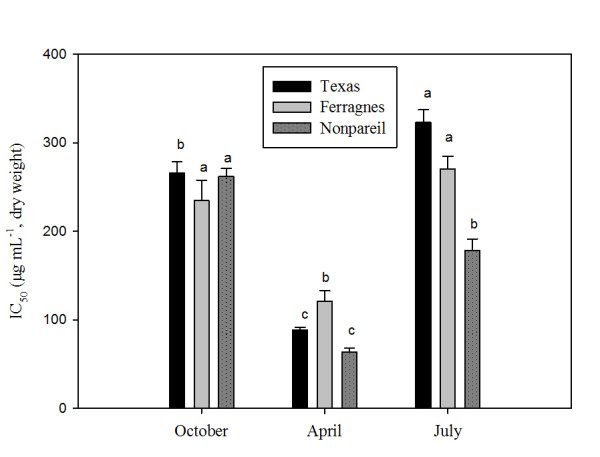
**Seasonal total IC**_**50**_ **changes in leaves of Nonpareil, Texas and Ferragnes in DPPH.** (Data followed by different letters are significantly different from each other (p < 0.05) according to Duncan’s test).

**Figure 2:**
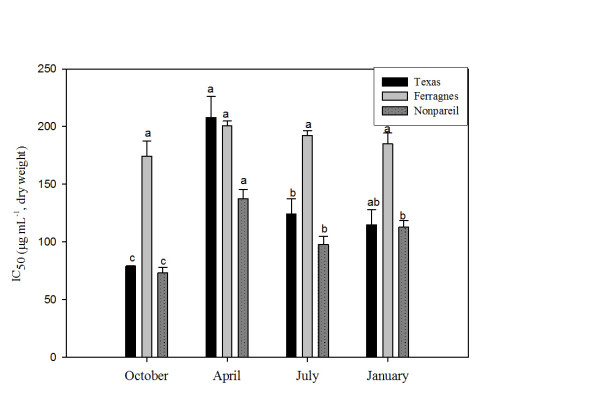
**Seasonal total IC**_**50**_ **changes in stems of Nonpareil, Texas and Ferragnes in DPPH.** (Data followed by different letters are significantly different from each other (p < 0.05) according to Duncan’s test).

It was found that total antioxidant activity varied according to season, plant organs and varieties (Figures 1 and 2). Total antioxidant capacity in the leaves of almond varieties (IC_50_) was low in April in Texas, Ferragnes and Nonpareil (high antioxidant activity) (Texas, 88.67 μg mL^-1^; Ferragnes, 121 μg mL^-1^; Nonpareil, 64 μg mL^-1^) (p < 0.05). The highest IC_50_ value (low antioxidant activity) was found in July for Texas, Ferragnes and in October for Nonpareil (Figure 1). It was determined that antioxidant capacity was the lowest for Nonpareil (high antioxidant activity) and high (low antioxidant activity) for Ferragnes in April (Figure 1) (p < 0.05).

IC_50_ values in the stems of almond varieties were low in October (high antioxidant activity) (Texas, 79.16 μg mL^-1^; Ferragnes, 174.46 μg mL^-1^; Nonpareil, 73.50 μg mL^-1^); and high in April (low antioxidant activity) (Texas, 207.79 μg mL^-1^; Ferragnes, 200.67 μg mL^-1^; Nonpareil, 137.67 μg mL^-1^) (Figure 2). The variation in antioxidant activity was significant in other varieties excluding Ferragnes (p < 0.05). IC_50_ values of Ferragnes and Texas varieties were similar in July and January. On the other hand, it was found that IC_50_ values were at the lowest level in Nonpareil and Texas (high antioxidant activity) and high in Ferragnes (low antioxidant activity) in October (Figure 2).

### Total phenolic compounds

**Figure 3:**
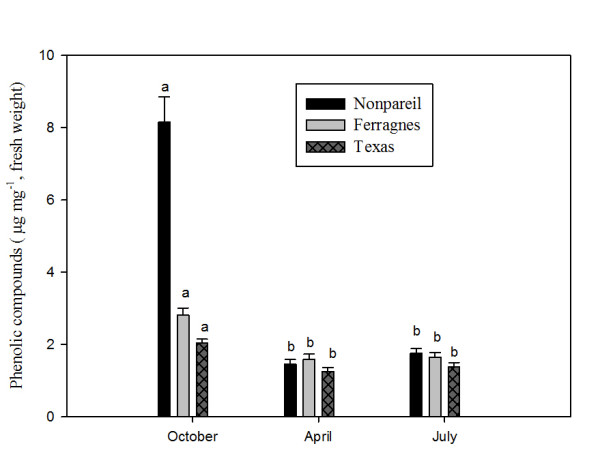
**Seasonal total phenolic compounds in leaves of Nonpareil, Texas and Ferragnes.** (Data followed by different letters are significantly different from each other (p < 0.05) according to Duncan’s test).

Phenolic compounds in the leaves of Nonpareil, Texas and Ferragnes varieties were high in October (Figure 3) (p < 0.05). In this month, values of phenolic compounds of Texas, Ferragnes and Nonpareil were 2.03 μg mg^-1^, 2.82 μg mg^-1^ and 8.15 μg mg^-1^ respectively. In all varieties, phenolic compounds were low in April and July and the variations observing in April and July were not significant statistically (Figure 3) (p > 0.05).

**Figure 4:**
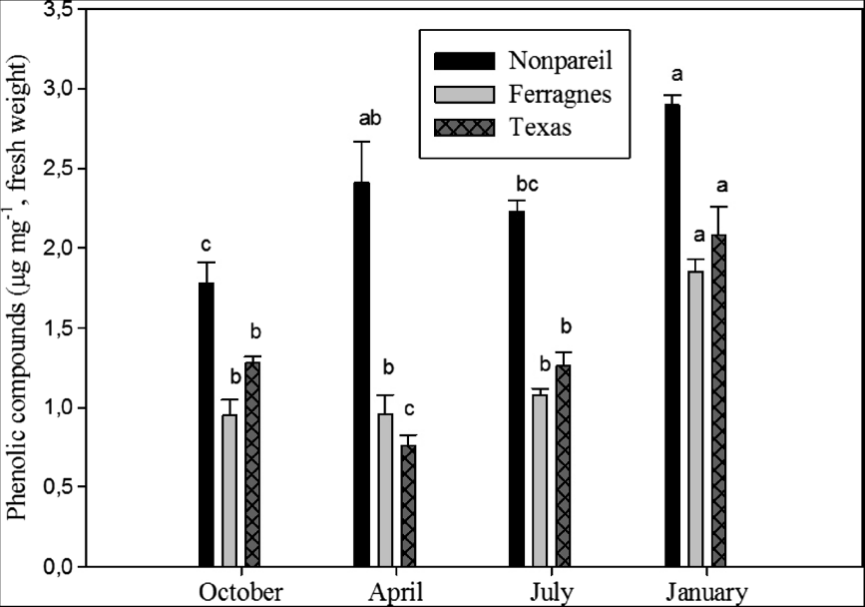
**Seasonal total phenolic compounds in stems of Nonpareil, Texas and Ferragnes.** (Data followed by different letters are significantly different from each other (p < 0.05) according to Duncan’s test).

It was found that phenolic compounds in the stems of almond varieties also varied according to months. In all varieties, phenolic compounds were the highest in January (Teksas, 2.08 μg mg^-1^; Ferragnes, 1.85 μg mg^-1^; Nonpareil, 2.90 μg mg^-1^) (Figure 4) (p < 0.05). The lowest phenolic compound contents were in October (0.95 μg mg^-1^) and July (1.08 μg mg^-1^) for Ferragnes and; in April for Texas (0.77 μg mg^-1^). In Nonpareil, levels of phenolic compounds were higher than other two varieties in all months (Figure 4).

## Discussion

Nunes et al. [24] carried out a study in red propolis and investigated the effect of season on antioxidant activity and total phenols. The researchers reported that there was a correlation between total antioxidant activity and season and that phenol content was high in hydra-alcoholic (90%) concentration in October. Ignacio et al. [25] reported that photosynthetic pigment and antioxidant activity in *Fagus sylvetica* L. varied by sun and light conditions.

In another study, carried out on different cultivars of California almonds, it was determined that flavonoid content and antioxidant activity depended on the cultivar rather than season [20]. As indicated above, this study found that antioxidant activity showed seasonal variations in stem and leaves of almond varieties (Nonpareil, Texas and Ferragnes) (Figures 1 and 2). Esfahlan and Jamei [26] carried out a study in fruits of ten wild almond species and reported that there were variations in flavonoid, phenolic contents and antioxidant activities according to almond species. The present study found that antioxidant activity varied according to varieties and plant organs. In April, antioxidant activity was the highest in the leaves of Nonpareil variety and the lowest in Ferragnes (Figure 1). On the other hand, in stems, it was high in Nonpareil and Texas and low in Ferragnes in October (Figure 2).

Cosmulescu and Trandafır [27] investigated the seasonal variation of total phenols in the leaves of *Juglans regia* L. They found that total phenols increased in June and July; decreased in August and increased in early September. They reported that there could be a correlation between phenolic content, season, genetic and ecological factors in walnut leaves. Sivaci and Sökmen [28] carried out a study on stem cuttings of *Morus alba* and *Morus nigra* and found that antioxidant activity and phenolic compounds showed seasonal variation. The highest antioxidant activity in stems was found in October.

In another study, variation of some phenolic compounds (phenylpropane chlorogenic acid and flavonoids such as rutin, hyperoside, epigenin-7-O-glucoside, kaempherole, quercitrin, quercetin and amentoflavone) in four *Hypericum triquetrifolium* populations in Central Black Sea Region were explored. Chemical variation was identified between the populations and plant sections and it was reported that these variations could be a result from different genetic, environmental and morphological factors [29]. In our study, total phenolic compounds varied according to season, variety and plant parts. The highest phenolic compound content in all varieties was observed in October in leaves; and in January in stems. The highest phenolic compound contents belonged to Nonpareil when compared to other varieties (Figures 3 and 4).

## Conclusions

It was found that total antioxidant activity and phenolic compounds in Nonpareil, Texas and Ferragnes varieties exhibited variations according to season, plant organ (leaf and stem) and variety. This could be result from ecological, genetic and metabolic differences as indicated other studies [27, 29]. Also, in the period during almond tree has no fruit, the leaves and stems could be made use of due to their antioxidant activity.

Further studies should be conducted to investigate the total antioxidant activity and phenolic profiles of almonds in next seasons.

## Methods

### Plant materials

Almond varieties (Nonpareil, Ferragnes and Texas) were collected from Lokman village of Adiyaman/Turkey (37° 42′ 15″ N, 38° 19′ 11″ E, 1920 feet) in 2011-2012. Leaves (April, July, October) and stems (April, July, October, January) of the almonds were analyzed. No analysis was performed in January because the plants had no leaves.

### Determination of antioxidant activity-DPPH

Leaf and stem samples collected from almond varieties were dried and grinded. Grinded samples were taken to methanol (MeOH) and extracted by shaking in water bath for 3 hours. Methanol extracts were then evaporated in evaporator under vacuum until they dried. Color of 2,2-diphenyl-1-picrylhydrazyl (DPPH) changes in the presence of antioxidant in the medium. Fifty microliters of various concentrations of almond variety extracts dissolved in methanol was added to in 5 mL of a 0.004% methanol solution of DPPH. The mixture was incubated at room temperature for 30 minutes and absorbance values were read at 517 nm [30]. Inhibition percent (I%) of DPPH was calculated according to the following equation:


where A_blank_ is the absorbance of the control reaction (containing all reagents except the test compound) and A_sample_ is the absorbance of the test compound. Inhibition is concentration dependent, and extract concentration providing 50% inhibition (IC_50_) is calculated from the graphplotted inhibition percentage against extract concentration. The assay was carried out in triplicate.

### Determination of total phenolic compounds

The leaf and stem samples were homogenized in 2.5 ml ethanol and shaken in water bath at 25°C for 24 h. Homogenized samples were filtered. 1 ml ethanol, 5 ml distilled water and 1 ml Folin-Ciocalteu reagent were added to 1 ml of the filtered samples and shaken well. After 3 minutes, 3 ml of Na_2_CO_3_ (2%, w/v) was added and shaken in a dark medium at intervals for 2 hours. Absorbance values were read at 760 nm for phenolic compound amounts and amounts were determined according to standard gallic acid equivalence [31, 32]. The assay was carried out in triplicate.

### Statistical analysis

All analyses in this study were performed in three replicates. SPSS version 15.0 was used for statistical analyses. Duncan tests were used to determine the variations between the means. Differences at 5% (p < 0.05) level were considered as significant.
